# Phenotypic Changes in T Cell and Macrophage Subtypes in Perivascular Adipose Tissues Precede High-Fat Diet-Induced Hypertension

**DOI:** 10.3389/fphys.2021.616055

**Published:** 2021-03-17

**Authors:** Ramya Kalyana Kumar, Yongliang Yang, Andres G. Contreras, Hannah Garver, Sudin Bhattacharya, Gregory D. Fink, Cheryl E. Rockwell, Stephanie W. Watts

**Affiliations:** ^1^Department of Pharmacology and Toxicology, Michigan State University, East Lansing, MI, United States; ^2^Department of Biomedical Engineering, Michigan State University, East Lansing, MI, United States; ^3^Institute for Quantitative Health Science and Engineering, Michigan State University, East Lansing, MI, United States; ^4^Department of Large Animal Clinical Sciences, Michigan State University, East Lansing, MI, United States; ^5^Institute for Integrative Toxicology, Michigan State University, East Lansing, MI, United States

**Keywords:** perivascular adipose tissue, sex-differences, immune cells, high-fat diet, hypertension, obesity, time course study

## Abstract

Perivascular adipose tissue (PVAT) may connect adiposity to hypertension because of its vasoactive functions and proximity to blood vessels. We hypothesized that immune cell changes in PVATs precede the development of high fat diet (HFD)-induced hypertension. Both sexes of Dahl S rat become equally hypertensive when fed a HFD. Further, both sexes would have similar immune cell composition in PVATs with the development and progression of hypertension. Male and female Dahl S rats were fed a regular (10% calories from fat; CD) diet or a HFD (60%) from weaning. PVATs from around the thoracic aorta (APVAT) and small mesenteric vessels (MRPVAT) were harvested at 10 weeks (pre-hypertensive), 17 weeks (onset), or 24 (hypertensive) weeks on diet. RNA-sequencing in MRPVAT at 24 weeks indicated sex-differences with HFD (>CD) and diet-differences in males (>females). The top 2 out of 7 immune processes with the maximum number of differentially expressed genes (DEGs) were associated with immune effector processes and leukocyte activation. Macrophages and T cells (and their activation status), neutrophils, mast, B and NK cells were measured by flow cytometry. Sex-specific changes in the number of CD4 memory T cells (males > females) and M2-like macrophages (females > males) in PVATs occur with a HFD before hypertension developed. Sex-differences became more prominent with the development and progression of hypertension, driven by the diet (HFD > CD). These findings suggest that though the magnitudes of increased blood pressure were equivalent in both sexes, the associated phenotypic changes in the immune subsets within the PVATs were different in the male vs. the female with the development and progression of hypertension.

## Graphical Abstract

Sex-differences in immune cell activation status (numbers/mean fluorescence intensity) in MRPVAT **(A)** and APVAT **(B)**. (Key: e.g., at 10 weeks, higher density/MFI of M1-like macrophages occur in CD females vs. CD males in MRPVAT.) Differences in mean arterial pressure between HFD and respective CD-fed rats are presented as mm Hg [Supplementary Figure 1 and as measured by radiotelemetry (Fernandes et al., 2018)] with 10, 17, and 24 weeks on diet.

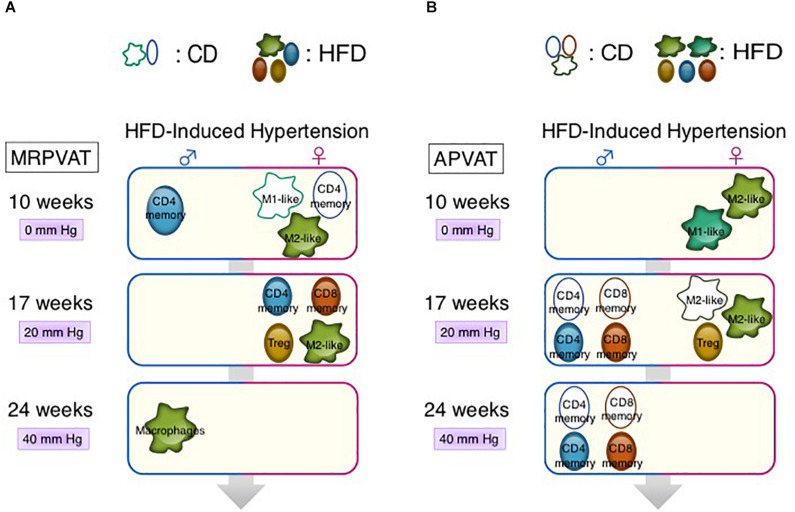

## Introduction

Over 45% of all adults in the United States have hypertension and up to 75% of those with hypertension are obese (Hall et al., 2015; Whelton et al., 2018). Both in obesity and hypertension, perivascular adipose tissue (PVAT), the fat around the peripheral blood vessels, loses its protective anti-contractile function partly due to local inflammation and oxidative stress (Szasz et al., 2013). Due to PVAT’s proximity to blood vessels and its functional capabilities, PVAT may be one of the links connecting obesity to hypertension (Gollasch, 2012; Noblet et al., 2015).

Perivascular adipose tissue contains multiple types of innate and adaptive immune cells in both sexes of healthy Sprague Dawley rats (Kumar et al., 2020). T cells may play a causal role in the development of hypertension in high salt-induced hypertension in male Dahl salt-sensitive (Dahl S) rats (De Miguel et al., 2010; Mattson et al., 2013). APVAT (PVAT around the thoracic aorta) inflammation and infiltration of macrophages and T cells precede atherosclerotic plaque development (another cardiovascular disease) (Skiba et al., 2017). This led us to hypothesize that immune cell infiltration in PVATs precedes the development of high fat diet (HFD)-induced hypertension. These immune cells in PVATs may directly (via cytokines) or indirectly (via other cells in PVATs/adipokines) alter vascular tone, thus leading to hypertension (Almabrouk et al., 2018; Nosalski and Guzik, 2017; Small et al., 2019).

Sex-differences in PVAT functions in obesity-induced hypertension (in association with resistin and estrogen) have been previously reported (Small et al., 2019; Taylor and Sullivan, 2016). Although T cell and macrophage counts increase in APVAT with AngII-hypertension in male mice, the occurrence of sex-differences in the immune system in PVATs with the development and progression of hypertension is little known (Mikolajczyk et al., 2016; Nosalski and Guzik, 2017). Both males and females of the Dahl S rat strain demonstrated increased adiposity and became equally hypertensive when fed a HFD (Fernandes et al., 2018). Hence, we also hypothesized that both sexes of Dahl S rats would have similar immune cell composition and activation status in PVATs prior to and with the development and progression of HFD-induced hypertension. Two types of PVATs in each sex were studied-MRPVAT (white adipose tissue-type PVAT around the first, second, and third order mesenteric resistance vessels only) and APVAT (brown adipose tissue-type around the thoracic aorta). Bulk RNA sequencing (MRPVAT) and flow cytometry (both PVATs) were employed to test the hypotheses. In support of our first hypothesis, we discovered that changes in specific subsets of T cells and macrophages in PVATs precede the development of HFD-induced hypertension. But contrary to our second hypothesis, clear sex-differences (females > males) in T cell and macrophage subsets were observed in both PVATs (MRPVAT > APVAT) prior to, and with the development and progression of hypertension. Although it is beyond the hypothesis and scope of this paper, understanding the vascular implications of these immune changes in PVATs is an important next step.

## Materials and Methods

### Animals

Animal maintenance and experimental protocols were approved by the MSU Institutional Animal Care and Use Committee and complied with the National Institutes of Health Guide for Animal Care and Use of Laboratory Animals (2011). Male and female Dahl salt sensitive rats were purchased from Charles River Laboratory at 3 weeks of age and were randomly fed a normal salt (0.3% NaCl) CD (D12450J, kcal from saturated fat 10% + carbohydrate, 70% + protein 20%) or a HFD (D12492, kcal from saturated fat 60% + carbohydrate 20% + protein 20%; Research Diets, Inc.). Animals were maintained on a 12/12 light/dark cycle at 22–25°C, fed on *ad libitum f*or 10, 17, or 24 weeks. Blood pressures were measured by tail cuff on multiple groups of rats that were used in the current study (Supplementary Figure 1). These measures correlate with blood pressures measured by radiotelemetry, as previously published by our group (Fernandes et al., 2018). Body weight and adipose tissue (including PVATs) masses are in Supplementary Figure 2.

### Bulk mRNA Sequencing: Sample Processing of MRPVAT

Males and females fed a CD or HFD for 24 weeks (5 per group) were used. RNA was extracted from MRPVAT using the Quick RNA MiniPrep kit (Cat# R1054) that includes a DNase step to remove genomic DNA. Purity, concentration, and integrity of mRNA were evaluated using a NanoDrop 1000 spectrophotometer (Thermo Scientific, Wilmington, DE, United States) and an Agilent Bioanalyzer 2100 system (Agilent Technologies, Santa Clara, CA, United States). All samples had a 260:280 nm ratio between 1.9 and 2.1 and RNA integrity number ≥ 7.5. Novogene Corporation Inc. (Sacramento, CA, United States) performed Quality Control for the RNA isolation that occurred at Michigan State University. Comparative libraries were constructed, library quality control was certified and sequencing was conducted.

### Flow Cytometry

Prior to all dissections, the overnight fasted rats were anesthetized with sodium pentobarbital (60–80 mg/kg, i.p.) and death was assured by creating a bilateral pneumothorax. MRPVAT and APVAT were dissected from blood vessels and weighed at 10, 17, or 24 weeks of diet, from each rat. All the immune cell populations were quantified from the same fat samples to phenotype the activation, differentiation and/or polarization of various immune populations.

#### Stromal Vascular Fraction Isolation From PVAT

The PVATs were minced with scissors and collagenase (1 mg/ml; type-I, Cat # LS004196) digested at 37°C for about 1 h. The cell suspensions were sequentially filtered through 100 and 40 μm filters. The flow through contained cells less than 40 μm, thus eliminating the adipocytes. Upon washing with flow buffer and centrifugation at 300 rcf for 5 min, a cell pellet containing the stromal vascular fraction (SVF) was obtained.

#### Surface Labeling of Immune Cells

Ammonium-chloride-potassium red blood cell lysis buffer (400 μL; Cat # 10-548E) was added to the SVF pellet, gently pipette-mixed and incubated on ice for 2 min to destroy red blood cells. The red blood cell-lysed SVF was washed twice with flow buffer (PBS + 10% FBS) and labeled with fluorescent antibodies (30 min incubation) after blocking Fc receptors with purified anti-CD32 antibody (10 min). Intracellular staining to identify Tregs was performed as previously described (Kumar et al., 2020). Viability was measured using Zombie-aqua dye (1:1,000 in dPBS, Cat# 77143). All flow cytometry assays were performed using an Attune NxT acoustic focusing cytometer from Life Technologies. Antibody and panel design details are in Supplementary Tables 1 and 2.

### Data Presentation and Statistics

#### Bulk mRNA Sequencing

mRNA isolated (at Michigan State University) from MRPVAT of both sexes fed a CD or a HFD were transcriptomic sequenced via Illumina platform by Novogene (Sacramento, CA, United States). After quality control, the paired-end clean reads were mapped onto reference genome using STAR (v2.5). HTSeq (v 0.6.1) was used to count the read numbers of each gene. The gene read counts matrix was fed into DESeq2 (v 1.24.0) to identify the differentially expressed genes (DEGs). To identify the outliers in the samples, principal component analysis was done with 10 principal components and the rotation matrix was used to calculate the Mahalanobis distance among the samples. To improve the stability of data, median (instead of mean) and mean absolute deviation around the median were used. For data analysis in R, tidyverse (v 1.3.0) and Rattus.norvegicus (v 1.3.1) libraries were used. For transcriptome analyses, the *p*-values were adjusted using Benjamini and Hochberg’s approach for controlling the false discovery rate. The RNA sequencing data were transformed into independent principal components (as many as the number of genes identified in a sample). The two principal components capturing the most variance in the data, PC1 and PC2, captured 64 and 24% of the whole variance. We can thus visualize the most important clustering patterns in the data using PC1 and PC2. Genes with an adjusted *P*-value < 0.05 and absolute fold change > 1, identified by DESeq2 were assigned as DEGs. To identify the immune system processes most affected by HFD or sex-differences, seven interpretable immune-system related Gene Ontology terms were chosen and the number of DEGs in each term was reported.

#### Flow Cytometry Studies

Gating of immune cell subtypes was performed as described in our previous publication (Kumar et al., 2020). Statistical analyses were performed with GraphPad Prism 8.0 (GraphPad Software Inc., La Jolla, CA). Two-way ANOVA with Tukey’s multiple comparison test was used to determine statistical significance. A *P* < 0.05 was considered statistically significant. Thus, two types of comparisons along the time course study were possible: between the sexes within each diet (with male as base) and between the diets within each sex (with CD as base).

## Results

### Bulk RNA-Sequencing Reveals Sex-Differences With HFD (>CD) and Diet-Differences in Males (>Females), in MRPVAT After 24 Weeks of Diet

RNA sequencing was performed on MRPVAT from male and female Dahl S rats on a CD or HFD for 24 weeks, a time point at which both sexes on a HFD were equally hypertensive vs. their respective CD rats (Supplementary Figure 1).

**FIGURE 1:**
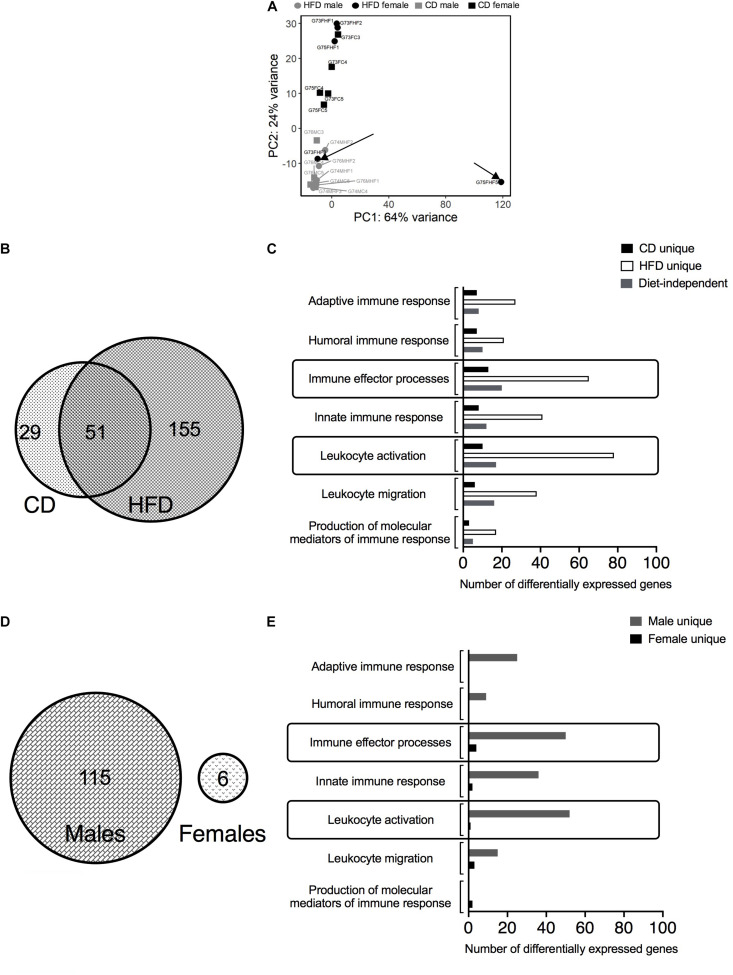
Bulk RNA-sequencing in MRPVAT of both sexes after 24 weeks on diet. Bulk RNA sequencing was performed on MRPVAT at 24 weeks on diet, from both sexes. Principal Component Analysis contained all the 20 samples (5 sample/diet/sex). **(A)** Venn diagram with the number of immune-related differentially expressed genes (DEGs) with respect to sex differences with HFD vs. CD **(B)** and the specific immune system processes affected **(C)**. Venn diagram with the number of immune-related DEGs with respect to diet differences in males vs. females **(D)** and the specific immune system processes affected **(E)**. G#: group number; M: male, F: female; C: CD; HF: HFD.

Principal components analysis (PCA) revealed the clustering patterns of the samples. G75FHF5 and G73FHF3 were statistical outliers. Hence, the remaining analyses excluded these animals. A clear separation of males from females along the PC2 axis suggested sex-differences in the transcriptomics (Figure 1A). As this study focuses on the immune cells in PVAT, only the immune system related DEGs were chosen. HFD induced more sex-differences vs. CD (155 vs. 29 unique DEGs). There were 51 shared DEGs between the diets, representing diet/blood pressure-independent (age-related) sex-differences in gene expression (Figure 1B). The top two (with the most number of DEGs) immune processes associated with the sex-differences influenced solely by the HFD-induced hypertension (“HFD unique”) were immune effector processes and leukocyte activation (Figure 1C). There were a greater number of diet-induced differences in males (115 DEGs) vs. females (6 DEGs). Also, there were no shared DEGs between the sexes, suggesting that the DEGs are strongly sex-dependent (Figure 1D). The top two unique immune processes associated with the diet-differences in males (“male unique”) were also immune effector processes and leukocyte activation. A similar conclusion could not be drawn for the females given the low number of unique DEGs (Figure 1E). The list of DEGs identified in Figures 1B,D and the associated R code used for analysis are available in the Supplementary Material as a spreadsheet and a.pdf file, respectively.

### Overall Changes in Innate and Adaptive Immune Profiles in PVATs With Progression of Hypertension in Males vs. Females

**FIGURE 2:**
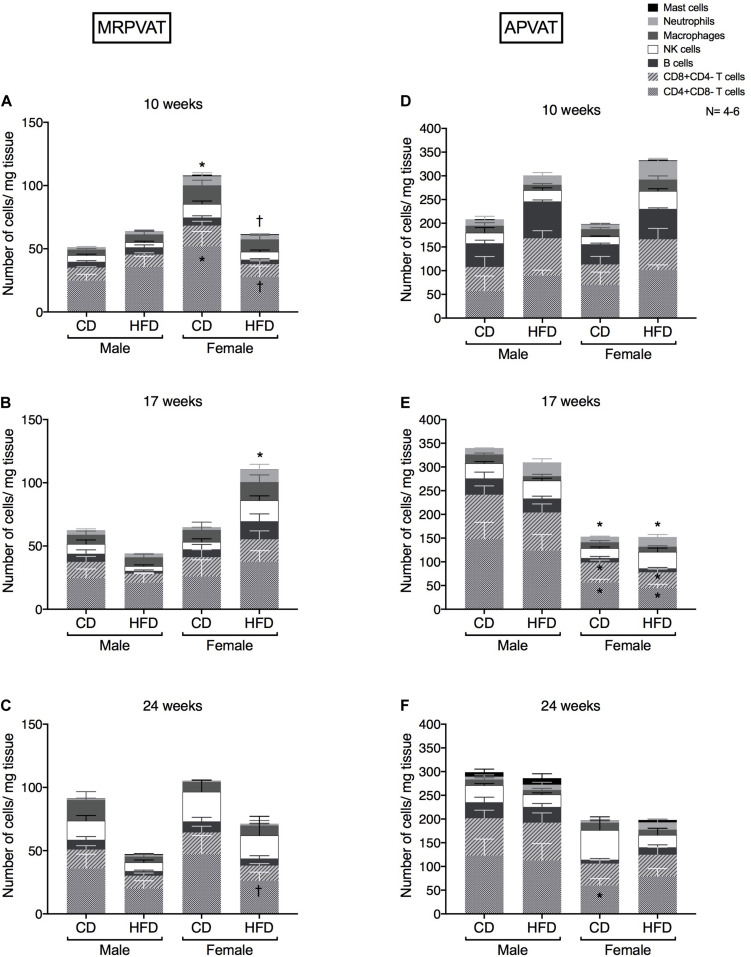
Overall snapshot of adaptive and innate immune cells in PVATs along the time-course of development and progression of hypertension. Immunophenotyping of mesenteric resistance PVAT (MRPVAT) and thoracic aortic PVAT (APVAT) in male and female Dahl S rats fed a control diet (CD) or a HFD for 10 **(A,D)**, 17 **(B,E)**, or 24 **(C,F**) weeks. Bars represent means ± SEM for the absolute cell counts of each immune cell type, normalized to tissue weight (in milligram). Number of animals is indicated by N. A *P* < 0.05 by two-way ANOVA with Tukey’s test for multiple comparison, was considered statistically significant. * and † inside the stacks in the graph represent a significant difference in the specific stack of immune cells between the sexes within each diet and between the diets within each sex respectively. * and † outside the stacks in the graph represent a significant difference in the sum total of immune cells between the sexes within each diet and between the diets within each sex respectively.

Six primary immune cell types: T cells (CD4 and CD8), B cells, macrophages, NK cells, mast cells, and neutrophils were quantified in MRPVAT (Figures 2A–C) and APVAT (Figures 2D–F), in both sexes after being fed a control diet (CD) or HFD for 10, 17, or 24 weeks. Corresponding blood pressure measures in Supplementary Figure 1. In the following figures, comparisons between the sexes within each diet (with male as base) and between the diets within each sex (with CD as base) were made.

#### MRPVAT

At 10 weeks, females on a CD had a greater number of CD4 T cells and sum of total immune cell counts vs. CD males and HFD females (Figure 2A). At 17 weeks, females on a HFD had a greater sum of total immune cell counts vs. CD females (Figure 2B). At 24 weeks, females on a HFD had a lesser number of CD4 T cells vs. CD females (Figure 2C). Although no diet-differences were observed in males, this finding is partially consistent with RNA seq data that diet-differences occur in females.

#### APVAT

There were no differences in the primary immune cell types between the sexes or diets (Figure 2D). At 17 weeks, females had reduced numbers of CD4, CD8 and sum of total immune cell counts vs. males, independent of the diet (Figure 2E). At 24 weeks, females on a CD continued to have a lesser number of CD4 T cells vs. CD males (Figure 2F). Males had no overall immune cell changes in PVATs at all time points of study. Also, APVAT in general has 3-7 times greater density of immune cells vs. MRPVAT.

T cells (dominant adaptive immune cell type in PVATs) and macrophage (dominant innate immune cell type in PVATs) became the focus to sub-characterize them as active/naïve/memory/effector phenotypes.

### At 10 Weeks, CD4 + Memory T Cell Counts Decrease in HFD Females (vs. Males) in MRPVAT While M1-Like Macrophage Numbers Increase in HFD Females (vs. Males) in APVAT Before Hypertension

**FIGURE 3:**
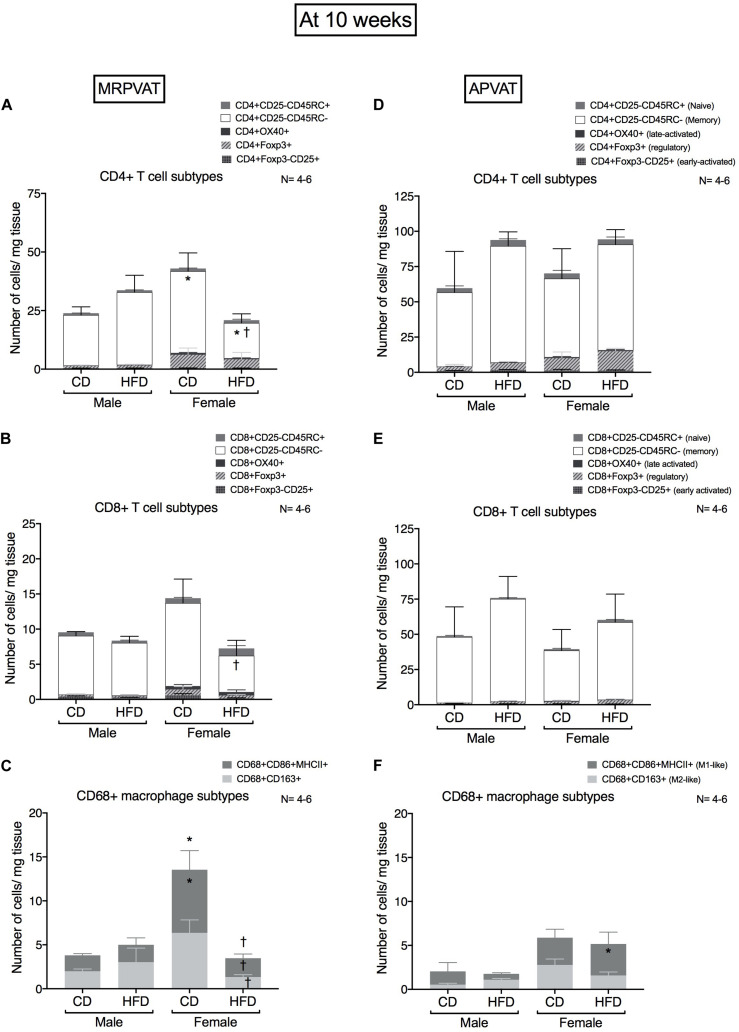
Sex-differences in T cell and macrophage subtypes in PVATs before the development of hypertension. Subtypes of CD4+, CD8 + T cells and CD68+ macrophages in MRPVAT and APVAT of both sexes at 10 weeks on diet (before hypertension) were quantified by flow cytometry. Subtypes of CD4 + : CD4 + CD25 +, CD4 + OX40 +, CD4 + Foxp3 +, CD4 + CD45RC- and CD4 + CD45RC + cells **(A,D)**, CD8 + T: CD8 + CD25 +, CD8 + OX40 +, CD8 + Foxp3 +, CD8 + CD45RC- and CD8 + CD45RC + cells **(B,E)**, and CD68 + CD68 + CD163 + and CD68 + CD86 + MHCII + macrophages **(C,F)** were measured. Bars represent means ± SEM for the absolute cell counts of each immune cell type, normalized to tissue weight (in milligram). Number of animals is indicated by N. A *P* < 0.05 by two-way ANOVA with Tukey’s test for multiple comparison, was considered statistically significant. * and † inside/outside the stacks in the graph represent a significant difference in the specific stack of immune cells/sum total of immune cells between the sexes within each diet and between the diets within each sex respectively.

Subtypes of CD4 T cells, CD8 T cells and macrophages were quantified in MRPVAT (Figures 3A–C) and APVAT (Figures 3D–F), after 10 weeks on a CD or HFD in both sexes by flow cytometry. CD4 and CD8 T cells were classified as recently activated (expressing early marker CD25 or late marker OX-40), regulatory (Foxp3+), antigen-experienced/memory (CD45RC-) or naïve (CD45RC+). CD68+ macrophages were classified as M1-like (expressing CD86 and MHCII) or M2-like (CD163+).

#### MRPVAT

Females on a CD had a greater number of CD4 memory T cells vs. males on a CD, while females on a HFD contained a reduced number of CD4 memory T cells vs. HFD males and CD females (Figure 3A). Females on a HFD had reduced CD8 T cell counts vs. females on a CD (Figure 3B). Females on a CD had a greater number of M1-like macrophages vs. males on a CD, while CD females had a greater number of both M1-like and M2-like macrophages vs. HFD females (Figure 3C).

#### APVAT

There were no differences in CD4 and CD8 T cell subtypes between the sexes or diets (Figures 3D,E). Females on a HFD had greater number of M1-like macrophages vs. males on a HFD (Figure 3F).

### At 17 Weeks, Immune Composition Changes Occur With the Onset of Hypertension That Is Mostly Diet-Dependent in MRPVAT (Females > Males) and Diet-Independent in APVAT

**FIGURE 4:**
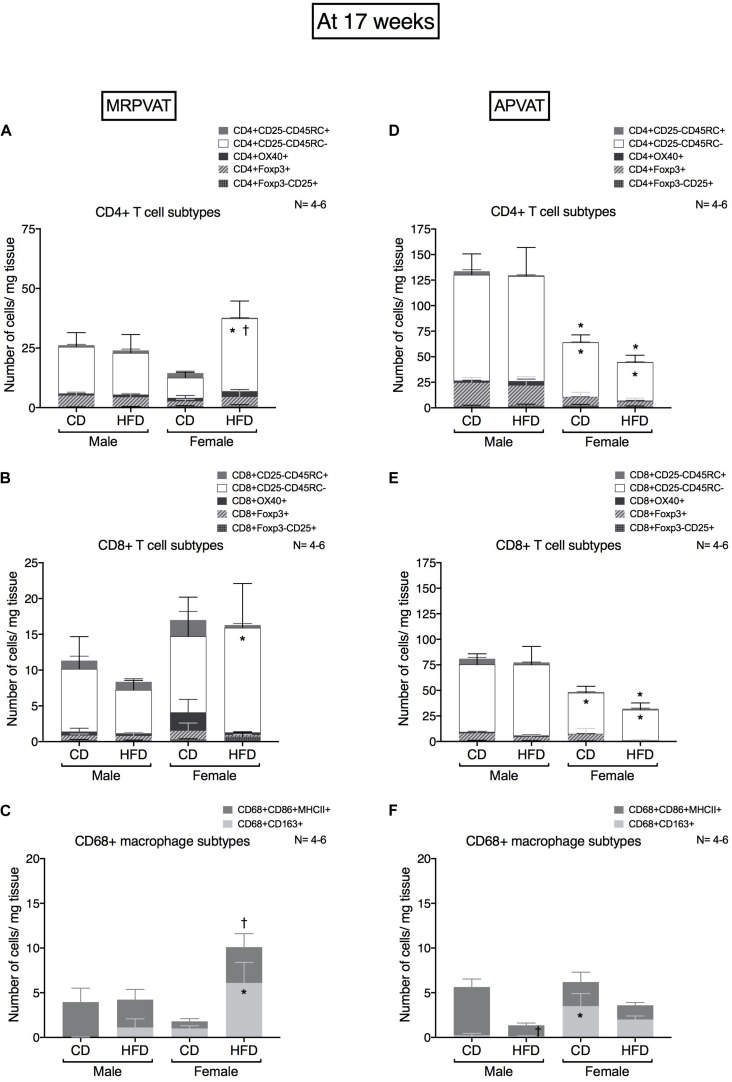
Sex-differences in T cell and macrophage subtypes in PVATs peak at 17 weeks on diet, with the onset of hypertension. Subtypes of CD4 +, CD8 + T cells and CD68 + macrophages in MRPVAT and APVAT of both sexes at 17 weeks on diet (onset of hypertension with HFD) were quantified by flow cytometry. Subtypes of CD4 + T cells **(A,D)**, CD8 + T cells: cells **(B,E)** and CD68 + macrophages **(C,F)** were measured. Bars represent means ± SEM for the absolute cell counts of each immune cell type, normalized to tissue weight (in milligram). Number of animals is indicated by N. A *P* < 0.05 by 2-way ANOVA with Tukey’s test for multiple comparison, was considered statistically significant. * and † inside/outside the stacks in the graph represent a significant difference in the specific stack of immune cells/sum total of immune cells between the sexes within each diet and between the diets within each sex respectively.

Subtypes of CD4 T cells, CD8 T cells and macrophages were quantified in MRPVAT (Figures 4A–C) and APVAT (Figures 4D–F), after 17 weeks on a CD or HFD in both sexes by flow cytometry.

#### MRPVAT

Females on a HFD had a greater number of CD4 memory T cells vs. HFD males and CD females (Figure 4A). Females on a HFD had a greater number of CD8 memory T cells vs. HFD males (Figure 4B). Females on a HFD had a greater number of M2 macrophages vs. HFD males (Figure 4C).

#### APVAT

Females had a lesser number of CD4 and CD8 memory T cells vs. males independent of the diet (Figures 4D,E). Females on a HFD had a greater number of M2-like macrophages vs. HFD males. Males on a HFD had a reduced M1 macrophage count vs. CD males (Figure 4F).

### At 24 Weeks, Diet-Dependent Immune Composition Changes With Hypertension in PVATs Begin to Occur in Males but Diminish in Females

**FIGURE 5:**
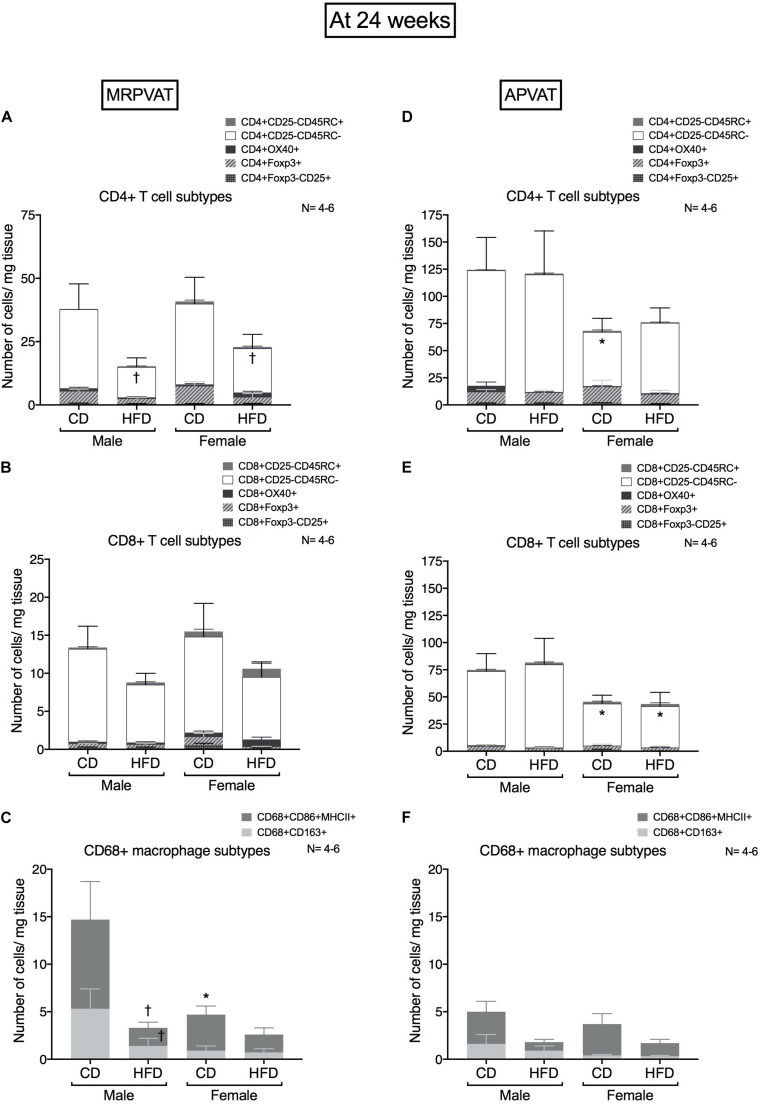
Sex-differences in immune cells diminish with the progression of hypertension after 24 weeks on diet. Subtypes of CD4 +, CD8 + T cells and CD68 + macrophages in MRPVAT and APVAT of both sexes at 24 weeks on diet (hypertensive with HFD) were quantified by flow cytometry. Subtypes of CD4 + T cells **(A,D)**, CD8 + T cells: cells **(B,E)**, and CD68 + macrophages **(C,F)** were measured. Bars represent means ± SEM for the absolute cell counts of each immune cell type, normalized to tissue weight (in milligram). Number of animals is indicated by N. A *P* < 0.05 by two-way ANOVA with Tukey’s test for multiple comparison, was considered statistically significant. * and † inside/outside the stacks in the graph represent a significant difference in the specific stack of immune cells/sum total of immune cells between the sexes within each diet and between the diets within each sex respectively.

CD4 T cell, CD8 T cell and macrophage subtypes were quantified in MRPVAT (Figures 5A–C) and APVAT (Figures 5D–F), after 24 weeks on a CD or HFD in both males and females by flow cytometry.

#### MRPVAT

High fat diet fed rats had a lesser number of CD4 memory T cells vs. CD fed rats independent of sex (Figure 5A). There were no differences in CD8 T cell subtypes between the sexes or diets (Figure 5B). Males on a HFD had a lesser number of M1-like macrophages vs. CD males (Figure 5C). This is consistent with the RNA seq results that diet-differences were prominent in males vs. females.

#### APVAT

Females on a CD had a reduced number of CD4 memory T cells vs. males on a CD (Figure 5D). Females had a reduced number of CD8 memory T cells vs. males, independent of diet (Figure 5E). There were no differences in macrophage subtypes between the sexes or diets (Figure 5F).

### Females on a HFD Have Higher Expression of M2-Like Macrophage (at 10 Weeks, Pre-hypertensive) and Regulatory T Cell Markers (at 17 Weeks, Onset of Hypertension) in PVATs vs. HFD Males

**FIGURE 6:**
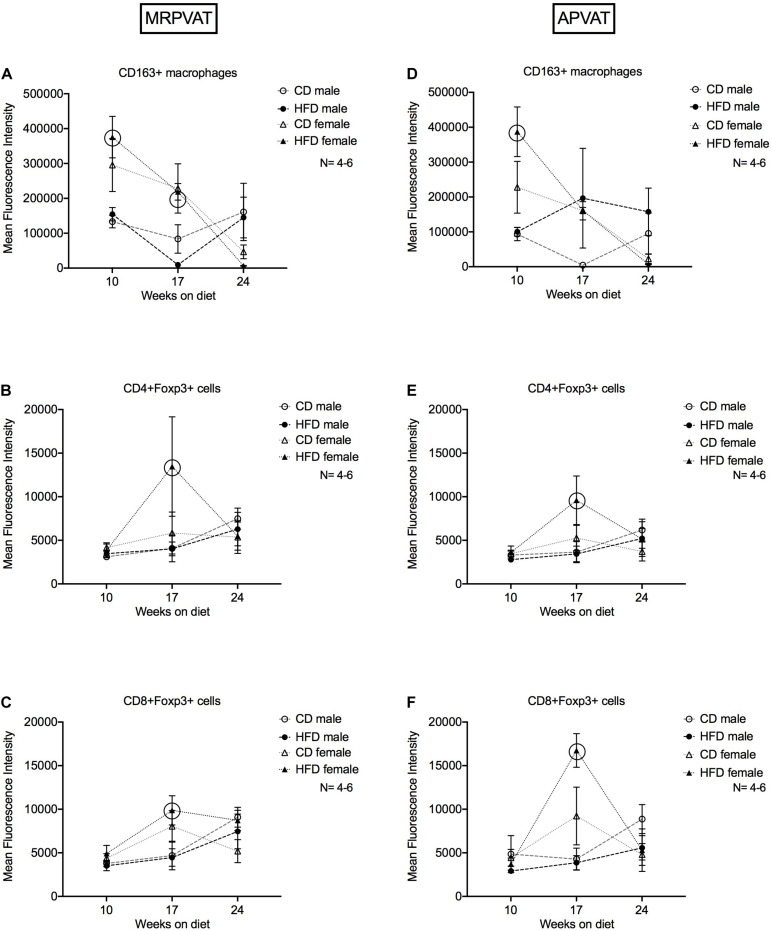
Magnitude of expression of markers of M2-like macrophages and T regulatory cells over the time course of hypertension development and progression. Mean fluorescence intensities (MFI) of CD163 on CD163 + M2-like macrophages **(A,D)**, Foxp3 on CD4 + **(B,E)** and CD8 + **(C,F)** T regulatory cells in MRPVAT and APVAT of both sexes over the time course on diet were quantified by flow cytometry. Points represent means ± SEM of the magnitude of expression (measured by MFI) of the specified marker. Number of animals is indicated by N. A *P* < 0.05 by two-way ANOVA with Tukey’s test for multiple comparison, was considered statistically significant. Circles in the graph represent a significant difference in MFI between the sexes within a diet at a given time point.

Mean fluorescence intensity (MFI) was used to quantify the magnitude of expression (different from absolute cell counts) of markers of specific immune cells over the time course of hypertension development and progression. MFI of all the markers used in the study were quantified. Only those that indicated sex or diet differences were included here. CD163 expression on CD163 + macrophages, Foxp3 expression on CD4+ Foxp3+ and CD8+ Foxp3 + T cells were quantified in MRPVAT (Figures 6A–C) and APVAT (Figures 6D–F), after 10, 17, or 24 weeks on a CD or HFD in both sexes by flow cytometry.

#### MRPVAT

Females on a HFD had greater expression of CD163 on CD163+ macrophages at 10 and 17 weeks vs. HFD males (Figure 6A). Females on a HFD had greater expression of Foxp3 on CD4 + Foxp3 + and CD8 + Foxp3 + T cells at 17 weeks vs. HFD males (Figures 6B,C).

#### APVAT

Females on a HFD had greater expression of CD163 on CD163+ macrophages at 10 weeks vs. HFD males (Figure 6D). Females on a HFD had greater expression of Foxp3 on CD4+ Foxp3+ and CD8+ Foxp3 + T cells at 17 weeks vs. HFD males (Figures 6E,F). Sex-differences with a HFD was prominent in terms of MFI, aligning with the RNA-seq data.

### Summary

Basal sex-differences in T cell and macrophage subtypes exist in PVATs in the Dahl S strain (at 10 weeks on a CD). After 10 weeks on HFD (before hypertension develops): sex-differences in T cell and macrophage subtypes were observed. Diet-dependent changes occurred in both males and females in a PVAT-specific manner. After 17 weeks on HFD (onset of hypertension): HFD-dependent changes were the most prominent in MRPVAT and diet-independent changes in APVAT. After 24 weeks on HFD (hypertensive): HFD-induced immune changes started to occur in males and diminish in females (also reflected in sequencing studies) in the PVATs (Graphical Abstract).

## Discussion

Treatment with the lymphocyte inhibitor mycophenolate mofetil lowers the increases in blood pressure in male Dahl S rats, suggesting a role for T and B cells in high salt diet-induced hypertension (Spradley et al., 2013). Mice lacking either functional macrophages or T and B cells resisted increases in BP with Ang-II treatment (Saely et al., 2012). Hence, immune cells are important causative links to hypertension development. The reason the current study focused on whether specific immune cell population changes in PVATs precede hypertension development is that such a finding would support immune changes as a cause rather than just an effect of hypertension. The primary goal of the current work was to understand whether immune cell changes in PVATs occurred prior to or after the development of hypertension. We hypothesized that such changes would be similar in the male and female, given that the magnitude and timeline of hypertension development was similar in Dahl S rats, when fed a high-fat diet (Supplementary Figure 1). Our findings suggested that the immune-level changes in PVATs preceded hypertension development but were different in the males vs. the females.

Only MRPVAT was chosen for the RNA-seq study because MRPVAT is a part of the visceral adipose tissue that is associated with increased risk for CVDs and MRPVAT surrounds arterial blood vessels that control peripheral vascular resistance (Saely et al., 2012). Thus, if immune-level changes were to occur in PVAT with hypertension, then MRPVAT (over APVAT) seemed to us as the apt choice for RNA-seq to get leads that would guide us to flow cytometry experiments focusing on specific immune cell types. The RNA-seq data at 24 weeks in MRPVAT support two main ideas. First, diet-differences in immune cell changes occurred in males (males > females) – this aligns with the flow cytometry data (Figures 1, 5). Second, sex-differences in immune changes in MRPVAT are more prominent with a HFD than a CD (particularly Figure 6). This is, however, in contradiction to the flow cytometry data that sex-differences diminish at 24 weeks. Flow cytometry panels are by necessity more limited in scope compared to the sequencing data. Thus, most of the gene expression changes that were revealed were not able to be assessed by flow cytometry. Also, cell numbers of a given type as measured by flow cytometry could diminish even if those cells expressed more of a specific set of genes as measured by bulk RNA-seq, making a direct comparison difficult.

### High Fat Diet-Induced Hypertension vs. Physiological Aging

Physiological aging is associated with increased visceral adipose tissue mass, systemic low grade inflammation and increased cardiovascular disease risk (Mau and Yung, 2019; Zemančíková and Török, 2019). All these phenotypes overlap with that of our experimental model.

Hence, effort was taken to tease out the gene expression differences (from the sequencing study) that were solely associated with a HFD vs. those that were “diet-independent or CD-dependent” or in fact “age-related.” Although sex-differences were not originally anticipated in this model, we confirmed findings from the sequencing data with the flow cytometry studies.

### Sex as a Biological Factor for Immunological Changes in PVAT: Causal for Development of Hypertension?

Sex-differences in the immune system functions, in PVAT functions and in the development and progression of hypertension are well-recognized in both humans and rodents (Small et al., 2019; Spradley et al., 2013; Tipton and Sullivan, 2014). In the current study, there were sex-differences in T cells and macrophage sub-populations in PVATs even at baseline. Females exhibit greater changes in immune populations (especially T cell and macrophage subtypes) with HFD-induced hypertension. This aligns with a previous finding that T cell profile in abdominal adipose tissue of females changes more with aging in mice vs. males (Spradley et al., 2013). T cells, by contributing to macrophage recruitment, may thus precede macrophage infiltration in obesity (Nishimura et al., 2009). Also, we observed an increase in memory T cells in MRPVAT (HFD-dependent and time point-dependent sex differences) and in APVAT (in males only and diet independent). Similarly, memory T cells increased in blood, vasculature and kidneys of hypertensive mice (Itani and Harrison, 2015). In our study, although there were no differences in the number of Tregs with hypertension in the sexes, females on a HFD with moderate increase in blood pressure vs. age-matched HFD males had increased expression of Foxp3 on Tregs, consistent with females maintaining more Tregs vs. males in Ang II hypertension and SHR (Pollow et al., 2014; Tipton and Sullivan, 2014).

In our study, although the blood pressure rises equally in both sexes with a HFD, the basal immunological differences in PVATs are exacerbated with HFD-induced hypertension in females more than males. These taken together suggest that any immune mechanisms in PVATs driving hypertension are different in males vs. females. This is also supported by findings from another study from our group, using the same Dahl S-HFD model. They demonstrated that only HFD-males developed blood pressure-associated renal inflammation and injury while the HFD-females seem to be protected against such renal injury by maintenance of the Treg population in the kidneys (Fernandes et al., 2018). Several reasons could explain the observed sex-differences in T cell and macrophage subtypes including the sex-hormones (Davis et al., 2014; Meyer et al., 2016; Taylor and Sullivan, 2016), cytokines (Henstridge et al., 2019; Tipton and Sullivan, 2014), adipokines (Considine et al., 1996; Landt et al., 1998), genetics/epigenetics (Mattson et al., 2008), and microbiota (Beale et al., 2019; Karbach et al., 2016) and thus pave ways for exciting future investigations.

### Are all the Immune Changes in PVATs Detrimental?

Macrophages and T cells were the top two immune cell types identified to play a role in the sex-differences in the development and progression of HFD-induced hypertension in the current study. HFD-fed mice had increased M1-like macrophage infiltration in APVAT (Almabrouk et al., 2018). Ang II increased proinflammatory monocyte/macrophage infiltration in PVAT in Treg-deficient mice (Mian et al., 2016). In the current study, a surge in the M1-like macrophage levels was observed at 10 weeks (CD females in MRPVAT and HFD females in APVAT), suggesting that immunological PVAT dysfunction precedes the development of hypertension. Although M2-like macrophages are in general protective, several studies point to the role of M2-like macrophages in dysfunctional AT, insulin-resistance (Wentworth et al., 2010) or vascular remodeling and fibrosis (Moore et al., 2015). However, at this point, we cannot interpret if the immune changes we observed are beneficial or detrimental, or neutral. Thus, deeper phenotypic characterization of the macrophage and other immune cell subtypes identified in the current study would be an excellent next step to understand their function.

### Physiological Relevance of Our Current Findings

APVAT had 3-7 times higher density of immune cells vs. MRPVAT in Dahl S rats (current study) and SD rats in both sexes (Kumar et al., 2020). This difference may be due to the differences in PVAT functions or the differences in the functions of blood vessels they surround. Aorta is a conduit vessel that stiffens during hypertension and the surrounding PVAT (APVAT) is brown-like fat. On the other hand, mesenteric resistance vessels are directly responsible for the control of total peripheral vascular resistance and the surrounding PVAT (MRPVAT) is white fat-like PVAT, part of the visceral fat that is associated with cardiovascular complications in obesity. Thus anatomically and physiologically APVAT is different from MRPVAT. In addition, our previous paper on healthy Sprague Dawley rats revealed that APVAT had a much greater density of immune cells vs. MRPVAT (Kumar et al., 2020), similar to our findings in the current paper on Dahl S rats. Based on these, we expected to see a higher immune cell density in APVAT vs. MRPVAT but could not hypothesize any further due to lack of studies directly comparing MRPVAT with APVAT.

Of course a key question is: Do immune cell changes in APVAT or MRPVAT (or both) promote hypertension in our animal model? Currently there are no PVAT-specific cells/proteins to which would allow us to genetically manipulate all PVATs or one PVAT location or type. Immunological changes in PVATs precede the development of CVDs such as HFD-induced hypertension (current study) and atherosclerosis. This is important to know because assessing PVAT function at an early stage has the potential to prevent the development of hypertension/other CVDs. Clinically, women are more prone to adverse drug reactions in all anti-hypertensive drug groups (Rydberg et al., 2018). Thus, gender-based and tissue (PVAT)-specific pharmacological treatments may reduce the dosage and limit drug toxicity and may also lead to better blood pressure control.

## Limitations

Dendritic cells and eosinophils (Withers et al., 2017) could not be included due to the lack of availability of reliable flow cytometry markers in the rat. The origin of the specific immune cell subtypes identified in PVATs with HFD over the time course is not interpretable from the current study. Factors known to be linked to sex-differences in hypertension-gonadal hormones, cytokines and adipokines or their receptor levels were not the focus of this study and were not measured. Also, estrous staging or ovariectomizing females were not done. RNA-sequencing at 10- and 17-week time points were not performed because the blood pressures were the most elevated at 24 weeks with a HFD. Female CD vs. HFD demonstrated a few DEGs which was unexpected given the blood pressure differences observed. However, the quality of the input RNA and sequencing studies eliminates this being an experimental error. Finally, the functional roles or consequences of the dynamic immune cell changes in PVAT warrants further studies. This is of real interest to the future studies in our group.

## Conclusion

This rigorous time course study identified that immune cells (especially T cell and macrophage subtypes) in PVATs respond to a HFD before hypertension occurs in both sexes of Dahl S rats. Additionally, sex-differences in PVATs exist at baseline and become more prominent, driven by a HFD with the development and progression of hypertension.

## Data Availability Statement

The datasets presented in this study can be found in online repositories. The names of the repository/repositories and accession number(s) can be found below: NCBI GEO; GSE165369.

## Ethics Statement

The animal study was reviewed and approved by the Michigan State University Institutional Animal Care and Use Committee and complied with the National Institutes of Health Guide for Animal Care and Use of Laboratory Animals (2011).

## Author Contributions

RK, AC, GF, CR, and SW contributed to the conception and design. RK, AC, and HG contributed to the acquisition of data. RK, YY, GF, CR, and SW contributed to the analysis and interpretation of the data. RK and SW involved in drafting of the manuscript. RK, YY, AC, SB, GF, CR, and SW involved in the manuscript revision and final approval. All authors contributed to the article and approved the submitted version.

## Conflict of Interest

The authors declare that the research was conducted in the absence of any commercial or financial relationships that could be construed as a potential conflict of interest.
