# Supplementary material for: Phenotypic Changes in T Cell and Macrophage Subtypes in Perivascular Adipose Tissues Precede High-Fat Diet-Induced Hypertension
Source: Front Physiol. 2021 Mar 17;12:616055. doi: 10.3389/fphys.2021.616055 (PMC8010306; doi:10.3389/fphys.2021.616055)
Supplement: Supplementary file 1 [file Table_1.docx]

**Supplementary Figure- S1**

**Figure S1: Development and progression of hypertension with high fat feeding.**

Comparison of mean arterial pressure (MAP) measured by tail cuff at 10, 17 and 24 weeks of control (CD) or high-fat (HFD) diet, in both males and females. Data are presented as mean+SEM. * denotes significant differences (p< 0.05; 2-way ANOVA with posthoc Tukey test) between CD and HFD within a sex at a given time point.

**Supplementary Figure- S2**

**Figure S2: HFD-fed animals have increased non-PVAT visceral fat mass.**

Body weights (A) weights of APVAT, MRPVAT and RP fat (retroperitoneal fat, non-PVAT white visceral fat) with 10 (B), 17 (C) and 24 (D) weeks on control (CD) or high-fat (HFD) diet. Tissue weights (in grams, g) are normalized to their respective body weights (in grams, g). * and † in the graph represent a significant difference (p< 0.05; 2-way ANOVA with posthoc Tukey test) in the specific tissue weight between the sexes within each diet and between the diets within each sex respectively.

**Supplementary Table- S1** List of antibody panels

| Antibody | Fluorochrome | Supplier | Clone | Dilution |
| --- | --- | --- | --- | --- |
| *Baseline* | | | | |
| Live/dead | Zombie aqua | Biolegend |  | 1:1000 |
| CD45 | APC/Cy7 | BD Bioscience | OX-1 | 1:200 |
| CD3 | FITC | BD Bioscience | 1F4 | 1:100 |
| *Immunophenotyping panel* | | | | |
| CD4 | BV605 | BD Bioscience | OX-35 | 1:50 |
| CD8a | PerCPeFluor710 | eBioscience | OX-8 | 1:100 |
| B220 | PE/Cy7 | BD Bioscience | OX-39 | 1:50 |
| CD68 | AF647 | Biorad | ED1 | 1:10 |
| CD161 | APC | Biolegend | 3.2.3 | 1:100 |
| HIS48 | FITC | BD Bioscience | HIS-48 | 1:100 |
| FcεRI | FITC | EMD Millipore | γ-subunit | 1:25 |
| Foxp3 | AF647 | Biolegend | 150D | 1:20 |
| *T cell panel* | | | | |
| CD4 | BV605 | BD Bioscience | OX-35 | 1:50 |
| CD8 | PerCPeFluor710 | eBioscience | OX-8 | 1:100 |
| CD25 | PE | BD Bioscience | OX-39 | 1:100 |
| CD134 | BV711 | BD Bioscience | OX-40 | 1:50 |
| CD45RC | AF680 | Santa Cruz | OX-22 | 1:50 |
| Foxp3 | AF647 | Biolegend | 150D | 1:20 |
| *Macrophage panel* | | | | |
| CD68 | AF700 | Biorad | ED1 | 1:10 |
| CD86 | AF647 | Biolegend | 24F | 1:50 |
| CD163 | PE | Thermofisher | ED2 | 1:50 |
| MHCII | BV711 | BD Bioscience | OX-6 | 1:50 |

**Supplementary Table- S2** Definitions of specific immune sub-populations

| Surface markers | Definitions |
| --- | --- |
| *T cell subtypes* |  |
| CD4+Foxp3-CD25+ or  CD8+Foxp3-CD25+ | Recently activated T cells (early marker) |
| CD4+OX40+ or  CD8+OX40+ | Recently activated T cells (late marker) |
| CD4+Foxp3+ or CD8+Foxp3+ | Regulatory T cells |
| CD4+CD25-CD45RC- or  CD8+CD25-CD45RC- | Memory (antigen experienced) T cells |
| CD4+CD25-CD45RC+ or  CD8+CD25-CD45RC+ | Naïve T cells |
| *Macrophage subtypes* |  |
| CD68+CD86+MHCII+ | Classically activated M1-like macrophages |
| CD68+CD163+ | Alternatively activated M2-like macrophages |
