# Supplementary material for: Phenotypic Changes in T Cell and Macrophage Subtypes in Perivascular Adipose Tissues Precede High-Fat Diet-Induced Hypertension
Source: Front Physiol. 2021 Mar 17;12:616055. doi: 10.3389/fphys.2021.616055 (PMC8010306; doi:10.3389/fphys.2021.616055)
Supplement: Supplementary file 3 [file Image_1.pdf]

```
# DEG analysis for Sex difference in PVAT tissues precede HF
# diet-induced hypertension

library(tidyverse)
library(Rattus.norvegicus)
library(DESeq2)


# 0. Define functions -----------------------------------------------------

DEG_cal <- function(cts, coldata, sam_list){
  ######## arguments:
  # cts expression readcount matrix
  # coldata: the meta data about samples
  # sam_list: the sample list for comparisons
  ######## return value
  # return a list of two items:
  # The DEGs with base level, Log2FC, and Padj
  # The # of DEGs for all comparisons

  DEG_list <- map(sam_list, function(x){
    cts_t <- cts[,x]
    coldata_t <- coldata[x,]
    dds_t <- DESeqDataSetFromMatrix(countData = cts_t,
                                    colData = coldata_t,
                                    design = ~ type)
    dds_t <- DESeq(dds_t)
    resMF <- results(dds_t, alpha = 0.05)
    resultsNames(dds_t)
    DEGs <- subset(resMF, padj < 0.05) %>% .[,c(1,2,6)]
    DEGs
  })
  list(DEG = DEG_list,
       DEG_n = map_dbl(DEG_list, nrow))
}


pca_ana <- function(dds_data, fn){

  ######## arguments:
  # dda_data: DESeq2 data structure
  # fn: the name and directory for saving the PCA plots
  ######## return value
  # no return value
```

```
  # save the PCA plot to the directory with the file name provided.

  vsd <- vst(dds_data, blind=FALSE)
  pcaData <- plotPCA(vsd, intgroup=c("gender", "type"), returnData=TRUE)
  percentVar <- round(100 * attr(pcaData, "percentVar"))

  pcaData %>% as_tibble(rownames = "sample") %>%
    ggplot(aes(PC1, PC2, shape = type, color = gender)) +
    geom_point(size=3) +
    xlab(paste0("PC1: ",percentVar[1],"% variance")) +
    ylab(paste0("PC2: ",percentVar[2],"% variance")) +
    ggrepel::geom_text_repel(aes(label = sample),
                             size = 2) +
    theme_classic()+
    scale_shape_manual(values=c(15, 16, 17, 18),
                       breaks=c("C", "HF"),
                       labels=c("Control", "High fat"))+
    scale_color_manual(values=c('#000000', '#999999'),
                       breaks=c("F", "M"),
                       labels=c( "Female", "Male"))+
    theme(
      text = element_text(size=14),
      legend.position = "top",
      panel.border = element_rect(colour = "black", fill=NA, size=1),
      legend.title = element_blank(),
      axis.title = element_text(size=14)
    )
  ggsave(fn, width = 6, height = 6, units = "in")
}


# 1. Load the readcount and Exploratory data analysis -------------------------

#readcount.csv: 18 sapmles. readcount_only_mrpvat.csv 20samples
read_count <- read_csv("../data/readcount_only_mrpvat.csv")
# with all the the samples for this study.
cts_with_outlier <- read_count[,-c(1:5)] %>% as.matrix()
rownames(cts_with_outlier) <- read_count[[1]]

#sample meta data
coldata_with_outlier <- tibble(
  samples = colnames(cts_with_outlier),
  type = map_chr(colnames(cts_with_outlier), str_sub,5,6),
  gender = map_chr(colnames(cts_with_outlier), str_sub,4,4),
```

```
  animal = map_chr(colnames(cts_with_outlier), str_sub,1,3)
)
coldata_with_outlier[c(15:20,6:9), 2] <- coldata_with_outlier[c(15:20,6:9), 2] %>% unlist %>%
str_sub(1,1)
coldata_with_outlier$type <-  factor(coldata_with_outlier$type, levels = c("C", "HF"))
coldata_with_outlier$gender <-  factor(coldata_with_outlier$gender, levels = c("F", "M"))

# remove the genes without gene symbols.
gene_ID_2_symbol <- select(Rattus.norvegicus, keys = rownames(cts_with_outlier),
columns="SYMBOL",
                    keytype="ENSEMBL") %>%
  .[complete.cases(.),] %>%
  .[!duplicated(.$ENSEMBL),] %>%
  as_tibble()
cts_with_outlier <- cts_with_outlier[gene_ID_2_symbol$ENSEMBL,]


# EDA
dds_with_outlier <- DESeqDataSetFromMatrix(cts_with_outlier,coldata_with_outlier,
                          design = ~ type)

# data distribution without normalization
boxplot(log10(counts(dds_with_outlier)+1))
#EDA after normalization
dds_with_outlier <- estimateSizeFactors(dds_with_outlier)
boxplot(log10(counts(dds_with_outlier,normalized=TRUE)+1))


fn <- "../results/dds_with_outlier.png"
pca_ana(dds_with_outlier, fn)


# we found two outlier, G75FHF5 and G73FHF3. The two outlier are in column 15 and 17 of the
read_count data frame
colnames(read_count)[c(17, 19)]

#Clearly G75FHF5 is an outlier, and we will run the DEG analysis using expression data matrix
with and without G73FHF3.

# 2. DEG analysis ----------------------------------------------------------

cts <- read_count[,-c(1:5, 17)] %>% as.matrix()
rownames(cts) <- read_count[[1]]
cts <- cts[gene_ID_2_symbol$ENSEMBL,]
```

```
# during PCA analysis, the "Type" is set as "HF" and "C".
# during DEG analysis, the "Type" should be set as "MHF", "MC", "FC", "FHF"
#sample meta data
coldata <- tibble(
  samples = colnames(cts),
  type = map_chr(colnames(cts), str_sub,4,6),
  gender = map_chr(colnames(cts), str_sub,4,4),
  animal = map_chr(colnames(cts), str_sub,1,3)
)

coldata[c(6:9, 14:19), 2] <- coldata[c(6:9, 14:19), 2] %>% unlist %>%   str_sub(1,2)
coldata$type <-  factor(coldata$type)
coldata$gender <-  factor(coldata$gender)
# the sample list for each experimental conditions with and with out G73FHF3
# sample. In the cts matrix G73FHF3 is in column 13
colnames(cts)[13]

sam_w_G73FHF3 <- list(
  HF_M_VS_F = c(1:5,10:13),
  Ctrl_M_VS_F = c(6:9, 14:19),
  Female_HF_VS_Ctrl = c(10:13,15:19),
  Male_HF_VS_Ctrl = c(1:9,14)
)

sam_wo_G73FHF3 <- list(
  HF_M_VS_F = c(1:5,10:12),
  Ctrl_M_VS_F = c(6:9, 14:19),
  Female_HF_VS_Ctrl = c(10:12,15:19),
  Male_HF_VS_Ctrl = c(1:9,14)
)

DEG_w_G73FHF3 <- DEG_cal(cts,coldata = coldata, sam_list = sam_w_G73FHF3)
DEG_wo_G73FHF3 <- DEG_cal(cts,coldata = coldata, sam_list = sam_wo_G73FHF3)

DEG_w_G73FHF3$DEG_n
# since the DEGs in FHF vs FC is zero with G73FHF3. Considering the significant
# effect of high fat diet, we removed G73FHF3 as an outlier.
# we further add another criteria as the absolute value of LOG2FC is larger than
# 1, which means the expressions in experimental groups are at least double
# or half of those in the control groups.

DEG_wo_G73FHF3 <- DEG_wo_G73FHF3$DEG
```

```
DEG_wo_G73FHF3_fc_1 <- map2(DEG_wo_G73FHF3, names(DEG_wo_G73FHF3), function(df,
nm){
  df <- df[abs(df[,2]) > 1,] %>% as_tibble(rownames = "Gene_ID")
  df$comparison <- nm
  df
})

DEG_wo_G73FHF3_df <- bind_rows(DEG_wo_G73FHF3_fc_1)
write_csv(DEG_wo_G73FHF3_df, "../results/DEG_gender_HF.csv")

# 2. identify the immune related DEGs ---------------------------------------

file_list <- list.files(path="../data/immunegotxtfiles")
file_name <-paste0("../data/immunegotxtfiles/", file_list)
GO_IMMU <- map(file_name, read_tsv, col_names = F)
names(GO_IMMU) <- str_sub(file_list, start =1  , end = 9)
GO_IMMU <- map(GO_IMMU, function(x){
  colnames(x) <- c("Gene_ID", "Symbol")
  x[[2]] <- toupper(x[[2]])
  x
})


# load the DEGs
DEG_gender_HF <- read_csv("../results/DEG_gender_HF.csv")
All_data <- DEG_gender_HF
# add the gene symbols
gene_ID_2_symbol <- select(Rattus.norvegicus, keys = All_data$Gene_ID, columns="SYMBOL",
                    keytype="ENSEMBL") %>%
  .[complete.cases(.),] %>%
  .[!duplicated(.$ENSEMBL),] %>%
  as_tibble()
gene_ID_2_symbol$SYMBOL <- toupper(gene_ID_2_symbol$SYMBOL)
All_data <- left_join(All_data, gene_ID_2_symbol, by = c("Gene_ID" = "ENSEMBL"))

comp <- unique(All_data$comparison)
comp_gender <- comp[1:2]
comp_diet <- comp[3:4]

# plot the venn diagram
venn_data <- function(comp1, fn){
  data_4_plot <- map(comp1, function(x){
    All_data %>% filter(comparison == x) %>%
      dplyr::select(SYMBOL) %>%
```

```
    unlist() %>% unname()
  })
  names(data_4_plot) <- comp1
  if(file.exists(fn)){
    print("file exists. Try another file name.")
  } else{
    venn.diagram(data_4_plot, fill = c("red", "green"),
                 filename = fn)
  }

  data_4_plot <- list(
    setdiff(data_4_plot[[1]], data_4_plot[[2]]),
    setdiff(data_4_plot[[2]], data_4_plot[[1]]),
    intersect(data_4_plot[[2]], data_4_plot[[1]])
  )
  names(data_4_plot) <- c(comp1, "shared")
  data_4_plot

}

fn_diet <- "../results/venn_diet.png"
fn_gender <- "../results/venn_gender.png"
comp_gender_res <- venn_data(comp_gender, fn_gender)
comp_diet_res <- venn_data(comp_diet, fn_diet)

names(comp_gender_res) <- c(paste0(names(comp_gender_res)[1:2], "_unique"),
                            paste0(names(comp_gender_res)[3], "_gender"))

names(comp_diet_res) <- c(paste0(names(comp_diet_res)[1:2], "_unique"),
                          paste0(names(comp_diet_res)[3], "_diet"))

fn_gender <- paste0("../results/", names(comp_gender_res), ".txt")
fn_diet <- paste0("../results/", names(comp_diet_res), ".txt")

walk2(comp_gender_res, fn_gender, write)
walk2(comp_diet_res, fn_diet, write)

## find the immune related DEGs


gene_2_immune <- function(genes){
  All_data_immu <- map2(GO_IMMU, names(GO_IMMU), function(x, name){
    genes <- intersect(genes, x$Symbol)
    dum_var <- All_data %>% filter(SYMBOL %in% genes)
```

```
    dum_var$GO_term <- name
    dum_var$Ratio <- nrow(dum_var) /  nrow(x)
    dum_var})
  All_DEG_immu <- All_data_immu[[1]] %>% filter(Gene_ID == 0)
  for(i in seq(length(All_data_immu))){
    All_DEG_immu <- bind_rows(All_data_immu[[i]], All_DEG_immu)}
  All_DEG_immu
}

comp_gender_res <- map(comp_gender_res, gene_2_immune)
comp_diet_res <- map(comp_diet_res, gene_2_immune)

DEGs_list <- list(comp_gender_res,
                  comp_diet_res
                  )

unique_immu_DEG <- function(DEGs_list){
  map(DEGs_list, function(DEGs){
    DEGs$SYMBOL %>% unique()
  })
}
comp_diet_res_DEG_list <- unique_immu_DEG(comp_diet_res)
comp_gender_res_DEG_list <- unique_immu_DEG(comp_gender_res)

fn_gender <- paste0("../results/", names(comp_gender_res), "_immune_related.txt")
fn_diet <- paste0("../results/", names(comp_diet_res), "_immune_related.txt")

walk2(comp_gender_res_DEG_list, fn_gender, write)
walk2(comp_diet_res_DEG_list, fn_diet, write)

# 3. identify the DEGs related the following 7 GO terms-----------------------

### the specific GO terms
GO_terms <- c(
  "GO0002440", # production of molecular mediator of immune response
  "GO0002250", # adaptive immune response
  "GO0045087", # innate immune response
  "GO0006959", # humoral immune response
  "GO0002252", # immune effector process
  "GO0045321", # leukocyte activation
  "GO0050900") # leukocyte migration

immue_DEG_numbers <- function(x){
```

```
  FC_smaller_0 <- x %>% filter(GO_term %in% GO_terms, log2FoldChange < 0) %>%
    group_by(GO_term) %>%
    summarise(
      FC_smaller_0 = n()
    )
  FC_larger_0 <- x %>% filter(GO_term %in% GO_terms, log2FoldChange > 0) %>%
    group_by(GO_term) %>%
    summarise(
      FC_larger_0 = n()
    )
  FC_count <- left_join(FC_smaller_0, FC_larger_0)
  FC_count[is.na(FC_count)] <- 0
  FC_count %>% mutate(
    Total = FC_smaller_0 + FC_larger_0
  )

}

comp_diet_immu <- map(comp_diet_res, immue_DEG_numbers )
comp_diet_immu$Female_HF_VS_Ctrl_unique$comp <- "Female_HF_VS_Ctrl_unique"
comp_diet_immu$Male_HF_VS_Ctrl_unique$comp <- "Male_HF_VS_Ctrl_unique"
comp_diet_immu$shared_diet$comp <- "Gender_indepent"
comp_diet_immu <- rbind(comp_diet_immu[[1]], comp_diet_immu[[2]],
comp_diet_immu[[3]])
write_csv(comp_diet_immu, "../results/selected_GO_terms_comparing_diet.csv")

comp_gender_immu <- map(comp_gender_res, immue_DEG_numbers )
comp_gender_immu$Female_HF_VS_Ctrl_unique$comp <- "Female_HF_VS_Ctrl_unique"
comp_gender_immu$Male_HF_VS_Ctrl_unique$comp <- "Male_HF_VS_Ctrl_unique"
comp_gender_immu$shared_diet$comp <- "Gender_indepent"
comp_gender_immu <- rbind(comp_gender_immu[[1]], comp_gender_immu[[2]],
comp_gender_immu[[3]])
write_csv(comp_gender_immu, "../results/selected_GO_terms_comparing_gender.csv")
```
